# Compact Full Ka-Band Waveguide Directional Coupler Based on Rectangular Aperture Array with Stairs

**DOI:** 10.3390/mi12070745

**Published:** 2021-06-25

**Authors:** Xida Deng, Ge Dong, Xuan Dai, Jinxiang Deng

**Affiliations:** 1 School of Aerospace Engineering, Tsinghua University, Beijing 100084, China; dxd20@mails.tsinghua.edu.cn; 2 School of Information and Electronics, Beijing Institute of Technology, Beijing 100081, China; 2702002011@bit.edu.cn; 3 School of Physics and Optoelectronics, Faculty of Science, Beijing University of Technology, Beijing 100124, China; jdeng@bjut.edu.cn

**Keywords:** compact directional coupler, microwave coupler, full bandwidth, 3 dB, rectangular aperture

## Abstract

This article presents a compact 3 dB waveguide directional coupler with full waveguide bandwidth. It consists of a pair of rectangular waveguides with stairs structures in the coupling region. The waveguides are placed parallel to each other along their broad wall, which has a rectangular aperture array. The compact size, broad bandwidth, good in-band coupling flatness, and good return loss are achieved by using the proposed structure. For verification purposes, a prototype of the proposed coupler was designed, manufactured, and measured. The experimental results show that over the full waveguide bandwidth a return loss of input port better than 17.46 dB, coupling strength varying between −2.74 dB and −3.80 dB, power-split unbalance within 0.76 dB, and an isolation better than 20.82 dB were obtained. The length of the coupling region was only 15.82 mm.

## 1. Introduction

Directional couplers are widely used in modern microwave and millimeter-wave applications such as radar systems, communication systems, and microwave imaging systems, which require power combining, power dividing, or power sampling. Waveguide structures are often used in high-frequency systems or high-power systems, because of their low insert losses and high-power handling characteristics.

As early as 1944, an example of waveguide directional coupler named the Bethe-hole coupler [1] was proposed. It has a single round hole on the common broad wall between a pair of rectangular waveguides. However, it is difficult to achieve broad bandwidth, tight coupling strength, and good coupling flatness. Some scholars proposed many new structures to improve the Bethe-hole coupler. The authors in [2] exhibit a cavity-type directional coupler using a simple structure that is easy to manufacture. In Reference [3], the Riblet coupler is presented, which consists of a short-slot hybrid junction. A simple compact narrow-wall directional coupler is developed in [4]. Multi-apertures are used in directional couplers in [5]. Moreover, multiport waveguide directional couplers are designed in [6].

In recent years, to satisfy the requirements of modern systems, many approaches have been presented to enhance coupling strength and enlarge bandwidth, including three coupling branch couplers [7], multilayer couplers [8], branch-line couplers [9], high-order directional couplers [10], and three-dimensional couplers [11]. These structures obviously expand the operation bandwidth. In Reference [12], a directional coupler using a printed ridge gap waveguide is proposed. Some new structures using substrate-integrated waveguide (SIW) are designed in [13,14,15,16,17]. Large apertures with transition structures [18] are presented to reduce the coupler size. They usually take several operating wavelengths to widen the bandwidth. It is difficult to achieve broad bandwidth or tight coupling strength with compact size.

**Figure 1:**
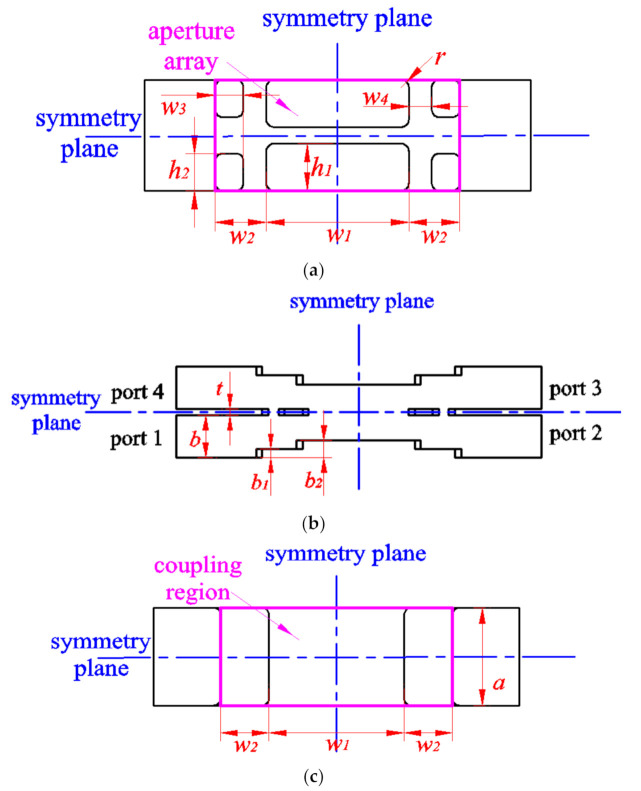

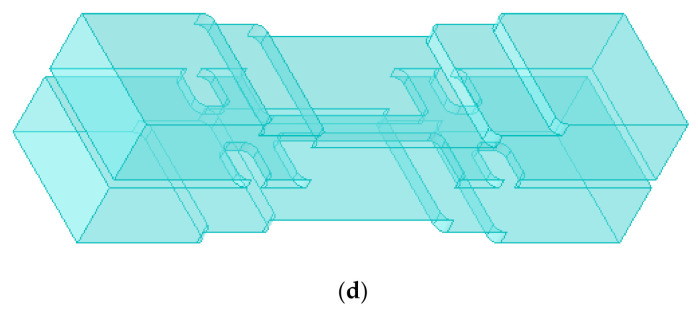
The proposed coupler: (**a**) rectangular aperture array, (**b**) side view, (**c**) top view, and (**d**) 3D-structure diagram.

The Ka band is a commonly used band for satellite communications, radar detection, and microwave imaging. In this article, we explain how coupling strength within −3.27 ± 0.53 dB over a full Ka band can be achieved simultaneously in compact size by using the novel rectangular aperture array with stairs, which are shown in Figure 1. The large rectangular aperture array (shown in Figure 1a) with floors (shown in Figure 1b,c) is first used in E-plane coupler. The 3D-structure diagram of the proposed coupler is shown in Figure 1d. This new structure can not only enhance the coupling strength effectively, but also reduce the size of the coupler. The floors (shown Figure 1b) can enhance the coupling strength and match the standard rectangular waveguide. Thus, this design can further decrease the size of the coupler. This structure can make the coupler size smaller than the coupler presented in [18]. The length of the coupling region (shown in Figure 1c) is only 15.82 mm, which is only 1.35 × λg (λg is operation wavelength of center frequency). A sample coupler was manufactured and measured and is described herein. The coupling coefficient is −3.27 ± 0.53 dB in the operation bandwidth varying from 26.5 GHz to 40 GHz, in which the return loss is better than 17.46 dB, and the isolation is higher than 20.82 dB. To the authors’ knowledge, no such compact 3 dB waveguide directional coupler with full waveguide bandwidth and good coupling coefficient has ever been presented before. The proposed coupler and some recently reported 3 dB directional couplers [11,18,19,20,21] are compared in Table 1.

## 2. Coupler Configuration

The configuration and port nominations of the proposed coupler, which includes three pairs of rectangular aperture arrays and the stairs structures placed in the coupling region of two rectangular waveguides, are shown in Figure 1. The main waveguide as well as the branch one are placed in parallel, and they are coupled to each other by three pairs of large aperture arrays in their common broad wall. Stairs structures are placed in the coupling region to enhance the coupling strength and to match the dimensions of standard rectangular waveguides (a = 7.12 mm, b = 3.56 mm).

The design results from the different numbers of rectangular apertures are listed in Table 2, with the bandwidth all assumed to be the same as the full WR-28 waveguide bandwidth, which is from 26.5 GHz to 40 GHz. The performance of the presented coupler is improved as the number of apertures and stairs increases. However, as the number of apertures increases, the length of the coupling region becomes longer. As a compromise, three pairs of rectangular aperture arrays are used in our prototype coupler.

**Figure 2:**
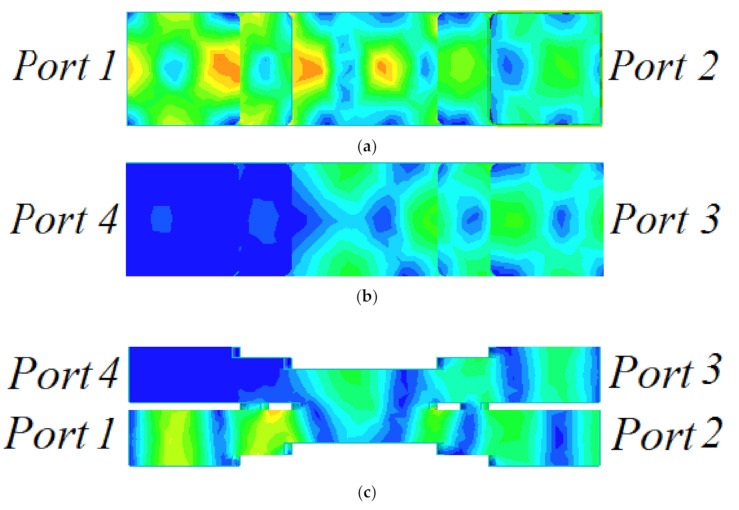
Surface current density at 33 GHz: (**a**) bottom view, (**b**) top view, and (**c**) side view.

TE_10_ mode is used in the proposed coupler. The coupling is less frequency dependent. The simulated surface current density is shown at 33 GHz in Figure 2. It can be observed that the signal, fed at port 1, is properly coupled at port 3, and port 4 is isolated. The surface current can flow into another waveguide across the large aperture array (Figure 1a), which is placed at the edge of the rectangular waveguide broadside. Further, the proposed structure reduces the size more significantly than the conventional designs.

**Figure 3:**
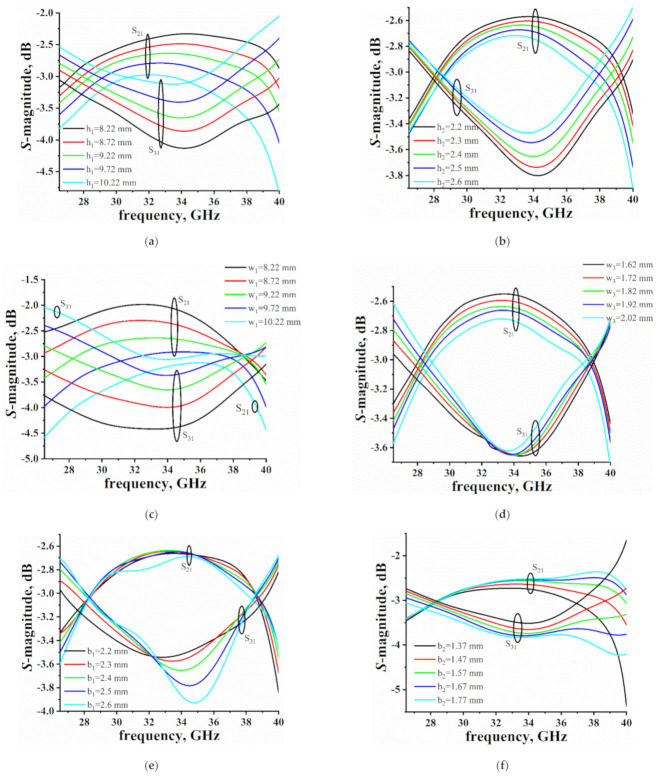
Simulated S_21_ and S_31_ magnitudes of the proposed coupler with: (**a**) varied h_1_ where the other dimensions are fixed as a = 7.12, b = 3.556, r = 0.5, t = 0.5, b_1_ = 0.71, b_2_ = 1.47, w_1_ = 9.22, w_3_ = 1.82, h_2_ = 2.4, and w_2_ = 3.3; (**b**) varied h_2_ where the other dimensions are fixed as a = 7.12, b = 3.556, r = 0.5, t = 0.5, b_1_ = 0.71, b_2_ = 1.47, w_1_ = 9.22, h_1_ = 3.03, w_3_ = 1.82, and w_2_ = 3.3; (**c**) varied w_1_ where the other dimensions are fixed as a = 7.12, b = 3.556, r = 0.5, t = 0.5, b_1_ = 0.71, b_2_ = 1.47, h_1_ = 3.03, w_3_ = 1.82, h_2_ = 2.4, and w_2_ = 3.3; (**d**) varied w_3_ where the other dimensions are fixed as a = 7.12, b = 3.556, r = 0.5, t = 0.5, b_1_ = 0.71, b_2_ = 1.47, w_1_ = 9.22, h_1_ = 3.03, h_2_ = 2.4, and w_2_ = 3.3; (**e**) varied b_1_ where the other dimensions are fixed as a = 7.12, b = 3.556, r = 0.5, t = 0.5, b_2_ = 1.47, w_1_ = 9.22, h_1_ = 3.03, w_3_ = 1.82, h_2_ = 2.4, and w_2_ = 3.3; (**f**) varied b_2_ where the other dimensions are fixed as a = 7.12, b = 3.556, r = 0.5, t = 0.5, b_1_ = 0.71, w_1_ = 9.22, h_1_ = 3.03, w_3_ = 1.82, h_2_ = 2.4, and w_2_ = 3.3 (all in millimeters).

Figure 3a–f illustrates the simulated S_21_ and S_31_ magnitudes of the proposed coupler with one parameter varied, where the other dimensions are fixed. Figure 3a,b shows the relationship between the heights of the aperture array and S_21_, S_31_ magnitudes. As h_1_, h_2_ become bigger, the S_21_ magnitude becomes smaller and the S_31_ magnitude becomes bigger. The higher frequency S parameter changes faster than the lower frequency S parameter. As is shown in Figure 3c,d, as w_1_ and w_3_ become bigger, the S_21_ magnitude becomes smaller and the S_31_ magnitude becomes bigger. The lower frequency is more sensitive to the width of coupling holes. In Figure 3e, we can see that the S_31_ magnitude is sensitive to b_1_ around 35 GHz. The higher frequency is more sensitive to b_2_ than the lower frequency, which is depicted in Figure 3f. It is found that there are two ways to change the coupling coefficient: one is to adjust the height of stairs (b_1_, b_2_) and the thickness of the common wall (t), while the other way is to change the parameters of the rectangular apertures (w_1_, h_1_, w_3_, and h_2_). The isolation of the coupler is influenced by the dimension of w_4_.

## 3. Coupler Design and Simulation Results

**Figure 4:**
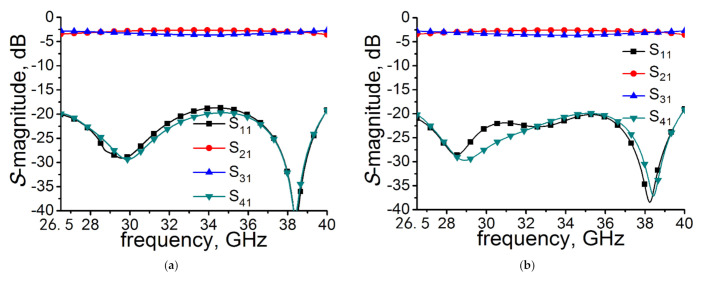
Simulated results of sample coupler (**a**) without transitions and (**b**) with transitions.

To show the design process of the compact coupler, a 3 dB coupler in full Ka band with novel rectangular aperture arrays and stairs was designed. The well-known commercial simulator HFSS (Ansys, Pittsburgh, Pennsylvania, U.S.) was used to simulate the coupler. Before the design process, we assumed that the structures of the two waveguides are the same as each other and placed parallel. The structure of the sample coupler is symmetric with the middle planes, which is shown in Figure 1. A perfect conductor was used to build the model and a discrete sweep type was used in the simulation. The process is described as follows. First, choose a standard rectangular waveguide (WR-28) (Guangsheng metal products company, Cangzhou City, Hebei Province, China) to meet the required operating frequency. Second, tune t, b_1_, b_2_, w_1_, h_1_, w_3_, and h_2_ (the size of rectangular holes and the height of floors) to make the coupling strength close to 3 dB. The coupling strength is determined by the size of rectangular coupling holes and the size of the height of floors simultaneously. If the rectangular holes become smaller, the height of floors must change to a bigger size. On the other side, if the rectangular holes become bigger, the height of floors must change to a smaller size. A compromise must be made between the size of rectangular holes and the height of floors. Then, adjust w_2_ to make the S_11_ magnitude better. Finally, a genetic algorithm is used to optimize t, b_1_, b_2_, w_1_, h_1_, w_3_, h_2_, and w_2_, and to make the S_21_ magnitude and S_31_ magnitude balance in the operating frequency. The dimensions of the variables depicted in Figure 1 were chosen as follows: a = 7.12, b = 3.556, r = 0.5, t = 0.5, b_1_ = 0.71, b_2_ = 1.47, w_1_ = 9.22, h_1_ = 3.03, w_3_ = 1.82, h_2_ = 2.4, and w_2_ = 3.3 (all in millimeter). The simulation curves are shown in Figure 4a, in which the S_11_ magnitude and S_41_ magnitude were below −18.68 dB and −19.15 dB, respectively, over full waveguide bandwidth. The S_21_ magnitude and S_31_ magnitude varied between −3.62 dB and −2.65 dB from 26.5 GHz to 40 GHz.

**Figure 5:**
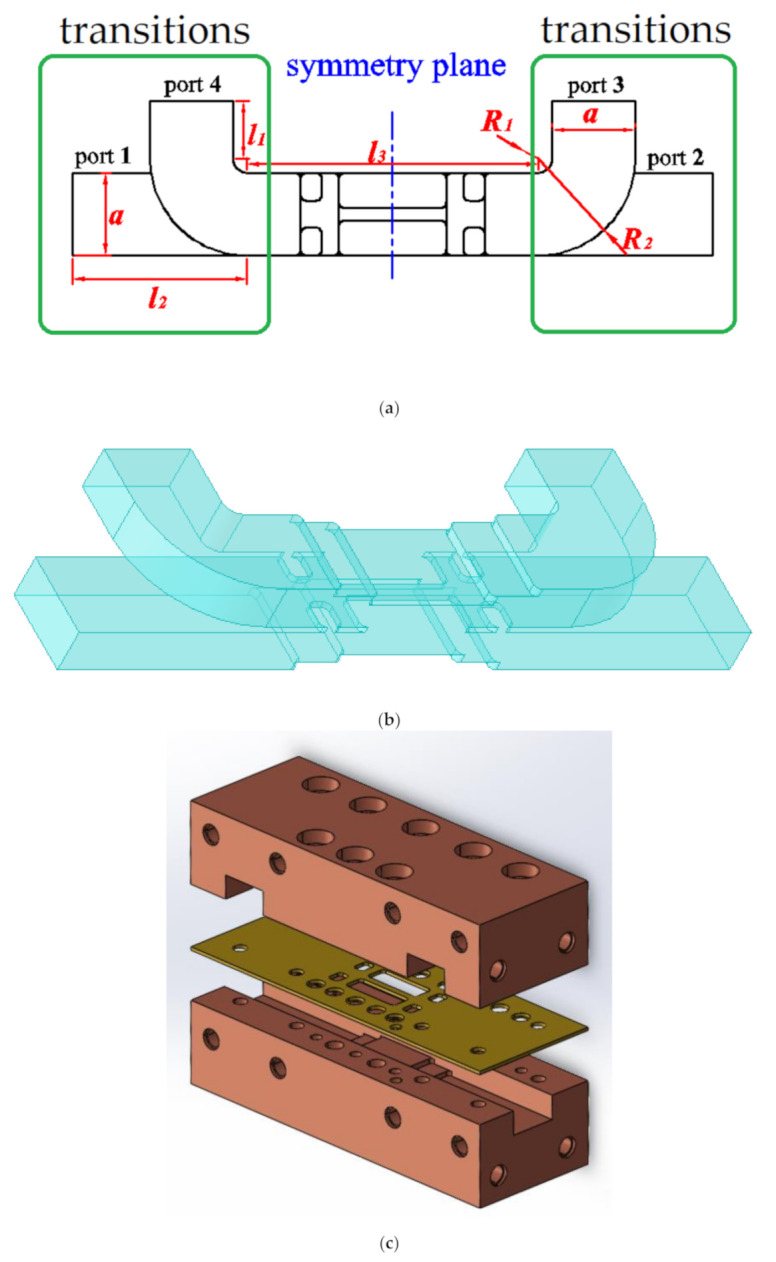
Prototype coupler (**a**) with transitions, (**b**) the 3D-structure diagram, and (**c**) exploded schematic.

For measurement purposes, two 90° transitions were added to the structure in order to facilitate the measurements. The dimensions of the coupler with transitions, which are shown in Figure 5a, are as follows: l_1_ = 5, l_2_ = 15, l_3_ = 25, R_1_ = 1.21, and R_2_ = 8.33 (all in millimeter). The simulated results of the coupler with transitions are depicted in Figure 4b, from which we can see that the S_21_ parameter and S_31_ parameter were almost unchanged compared with the no transitions situation, which is shown in Figure 4a, and the simulated S_11_ parameter and S_41_ parameter were below −18.97 dB and −19.16 dB, respectively, in the full Ka band. Figure 5b shows the 3D-structure diagram of the prototype coupler. The exploded schematic of the coupler is depicted in Figure 5c.

## 4. Results

### 4.1. Fabrication

**Figure 6:**
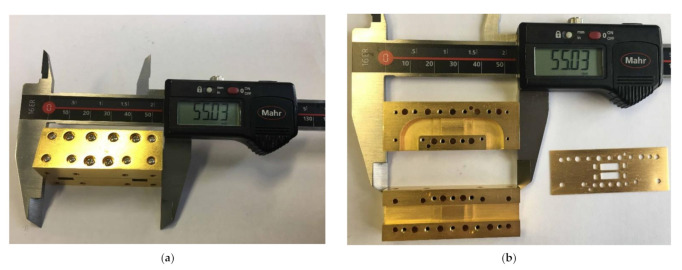
Photograph of sample coupler (**a**) assembled and (**b**) disassembled (the dimensions of sample coupler are 55.03 × 20.02 × 25.02 mm^3^).

The 3 dB full Ka band waveguide directional coupler based on rectangular aperture arrays with stairs is fabricated according to the configuration parameters given above and assembled/disassembled pictures are shown in Figure 6. The coupler is fabricated by a brass nut, gold plate.

### 4.2. Measurement Results

**Figure 7:**
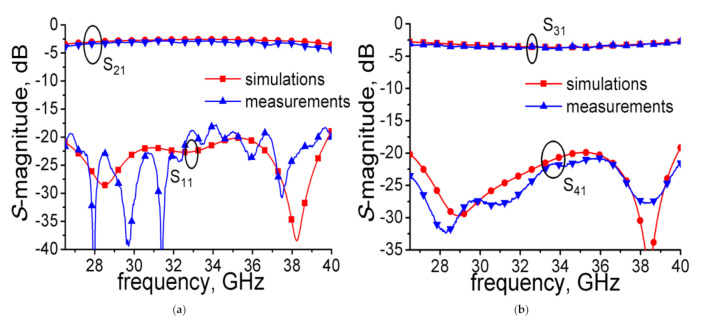
Simulated and measured results of the sample coupler: (**a**) S_11_ and S_21_ and (**b**) S_31_ and S_41_.

The measured results were performed using an Agilent N5224A (Keysight Technologies, Santa Rosa, California, U.S.) network analyzer. The calibration method used is the Short-Load-Open-Thru (SLOT) method. The measured results and simulated results are shown in Figure 7. Over the full waveguide bandwidth from 26.5 GHz to 40 GHz, the measured S_11_ magnitude and S_41_ magnitude were below −17.46 dB and −20.82 dB, respectively. The S_21_ magnitude varied from −2.89 to −4.26 dB, while coupling strength (S_31_-magnitude) changed from −2.74 to −3.80 dB. A power-split unbalance within 0.76 dB was achieved. The simulated and measured results of S_21_ and S_31_ are in good agreement. Some deviations between them may be caused by unexpected tolerances in fabrication, inaccurate material parameters, or loss of electromagnetic energy. The difference between simulation and measurement of S_11_ may be caused by imperfect parameters of the transitions and loads used in the test, and the difference of S_41_ may be caused by testing with no transitions and loads. The proposed coupler has a better performance in coupling flatness using smaller sizes compared with the couplers [5,6,7,8]. The conventional ones (coupling region) need several λg (λg is operation wavelength of center frequency) to achieve the performance shown in Table 1, while the sample coupler (coupling region) only needs 1.35 × λg (15.82 mm) to realize tight coupling strength, full waveguide bandwidth, and better coupling flatness. The overall sizes of the coupler are 55.03 × 20.02 × 25.02 mm^3^.

### 4.3. Sensitivity Analysis

**Figure 8:**
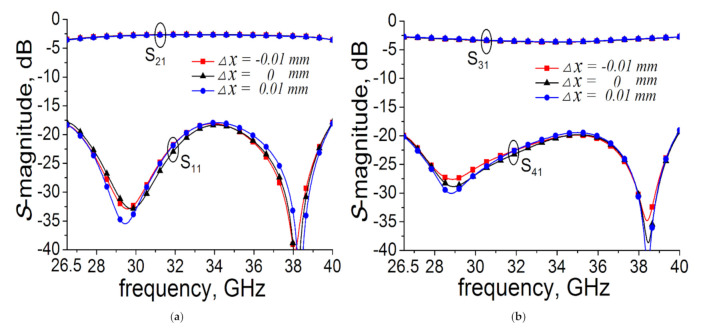
Sensitivity analysis: (**a**) S_11_ and S_21_ and (**b**) S_31_ and S_41_.

Unexpected tolerances may occur during the fabrication process. The margin of machining error is within ±0.01 mm. To illustrate the effect of these machining errors, we simulated the S magnitude using key structure parameters (b_1_, b_2_, w_1_, h_1_, w_3_, and h_2_) with errors, which are shown in Figure 8 (△x = −0.01 mm: b_1_ = 0.7, b_2_ = 1.46, w_1_ = 9.21, h_1_ = 3.02, w_3_ = 1.81, and h_2_ = 2.39; △x = 0 mm: b_1_ = 0.71, b_2_ = 1.47, w_1_ = 9.22, h_1_ = 3.03, w_3_ = 1.82, and h_2_ = 2.4; △x = 0.01 mm: b_1_ = 0.72, b_2_ = 1.48, w_1_ = 9.23, h_1_ = 3.04, w_3_ = 1.83, and h_2_ = 2.41 (all in millimeters)). As it can be observed, this design is very robust.

## 5. Conclusions

In this paper, a compact full Ka-band waveguide directional coupler is presented, which has good coupling flatness and broad bandwidth. Over the full Ka band from 26.5 GHz to 40 GHz, the S_11_ magnitude and S_41_ magnitude were below −17.46 dB and −20.82 dB, respectively. The S_21_ magnitude varied from −2.89 to −4.26 dB, while coupling strength (S_31_-magnitude) changed from −2.74 to −3.80 dB. A power-split unbalance within 0.76 dB was achieved. The length of the coupling region is only 15.82 mm. The overall sizes of the coupler are 55.03 × 20.02 × 25.02 mm^3^. The novel coupler can be easily designed to other frequency ranges and different coupling coefficients by following the design process given above. The proposed coupler could be a good candidate for miniaturized microwave system applications such as radar systems, communication systems, and microwave imaging systems. Based on this article, the research of deformable directional coupler, terahertz directional coupler may be carried out in the future.

## 6. Patents

The coupler proposed in this article has obtained Chinese patent protection, the patent number is ZL 2020 1 0225946.3.

## Figures and Tables

**Table 1:** Performance comparison of some recently reported 3 dB directional couplers.

Coupler	Frequency(GHz)	Coupling Strength Imbalance (dB)	Bandwidth (%)	Relative Length	Type
[11]	31–36.7	±0.54	16.8	1.41 × λg	SIW
[18]	6.57–9.99	±0.6	41.3	3.37 × λg	RW
[19]	10.675–13.325	±0.5	22.08	1.66 × λg	HMSIW
[20]	13–17	±0.5	26.67	1.41 × λg	ESIW
[21]	33–37	±0.33	11.43	0.71 × λg	SIW
This work	26.5–40	±0.53	40.6	1.35 × λg	RW

HMSIW: half-mode substrate-integrated waveguide. RW: rectangular waveguide. ESIW: empty substrate-integrated waveguide. SIW: substrate-integrated waveguide. λg: operation wavelength of the center frequency.

**Table 2:** Performance of 3 dB directional coupler with different number of large apertures.

Number of Apertures	Coupling Imbalance (dB)	Through Flatness (dB)	Return Loss (dB)	Coupler Model
2×1	3.17 ± 0.92	3.4 ± 1.2	15.19	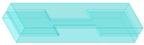
2×2	3.11 ± 0.7	3.23 ± 0.89	17.65	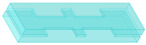
2×3	3.14 ± 0.46	3.13 ± 0.48	18.68	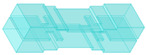

## Data Availability

Data is contained within the article.
